# The Agreement Between Reverse Transcriptase-Polymerase Chain Reaction (RT-PCR) and Rapid Antigen Test (RAT) in Diagnosing COVID-19

**DOI:** 10.7759/cureus.29266

**Published:** 2022-09-17

**Authors:** Rupesh Sahu, Amarnath Gupta, Sumit Rawat, Abhijit Das

**Affiliations:** 1 Preventive Medicine, Government Chhindwara Institute of Medical Sciences, Chhindwara, IND; 2 Preventive Medicine, Government Bundelkhand Medical College, Sagar, IND; 3 Microbiology, Government Bundelkhand Medical College, Sagar, IND

**Keywords:** sars-cov-2 testing, sars-cov-2 (severe acute respiratory syndrome coronavirus -2), coronavirus disease, covid 19, covid-19 retro, rat, rt-pcr, agreement, covid-19 diagnosis, sars-cov-2

## Abstract

Background

False-negative results derived from RT-PCR tests for diagnosing coronavirus disease (COVID-19) have raised questions about whether to consider them the gold standard for the detection of severe acute respiratory syndrome coronavirus 2 (SARS-CoV-2). Using an imperfect gold standard to assess other diagnostic tests would never let the other tests show better diagnostic performance. The best strategy in such cases is to do an agreement analysis, and this study aims to estimate the agreement between real-time reverse transcriptase-polymerase chain reaction (RT-PCR) and rapid antigen test (RAT) for COVID-19 detection.

Methods

A retrospective study was done using paired data of individuals tested for COVID-19, both by RT-PCR and RAT, obtained from the virology laboratory of Government Bundelkhand Medical College, Sagar, Madhya Pradesh, India. A sample size of 93 was calculated, and the data were abstracted in a data abstraction sheet. Variables included were results of RT-PCR and RAT, age, gender, presence of symptoms, test kit used, and the time duration between sampling for RT-PCR and RAT. Apart from descriptive statistics, keeping in mind the binary outcome of RT-PCR and RAT, Cohen’s kappa was calculated for agreement analysis. A p-value of <0.05 was considered significant.

Results

The data on 100 participants suspected to be infected with COVID-19 (58 male and 42 female) with a mean age of 39.8 (±19.0) years were analysed. The number of discordant pairs was eight. Cohen’s kappa showed substantial agreement between RT-PCR and RAT, κ=0.646, (95% CI 0.420 to 0.871), p<0.001.

Conclusion

Considering the ease of conducting RAT with quick results and substantial agreement with RT-PCR, RAT could be a better choice in detecting SARS-CoV-2 and, hence, COVID-19 disease on a large scale.

## Introduction

Rapid and accurate diagnostic tests are crucial for controlling any communicable disease; the same goes for the coronavirus disease (COVID-19) pandemic. Quantitative reverse transcriptase-polymerase chain reaction (RT-PCR) is recommended by the World Health Organization (WHO) as the reference test for the laboratory diagnosis of COVID-19 [1]. Researchers have developed other forms of diagnostic tools, such as antigen-detection diagnostic tests. Generally, the ease-of-use and rapid turnaround time of antigen-detecting rapid diagnostic tests (RDTs) offer to decrease the delays in diagnosis by shifting to decentralised testing of patients with early symptoms [2]. The Indian Council of Medical Research (ICMR) published an advisory regarding the use of the rapid antigen test (RAT) on June 14, 2020, and since then it has been used extensively [3].

No test is expected to produce 100% accurate results. Ideally, a new test is compared with an existing "gold standard" and parameters like sensitivity, specificity, and predictive values of the new test are calculated. There are reports of false-negative RT-PCR results in patients with COVID-19 during the pandemic course [1]. An early study [4] showed that the sensitivity of RT-PCR varies from 68% to 100%, and the specificity is 98.9%, but several authors [5,6] have pointed out the poor performance of this technique, particularly in terms of sensitivity. According to Liu et al., the positivity rate of RT-PCR could be as low as 38% [5] (i.e., not better than chance). The accuracy of the result depends on the site and the quality of sampling as well. Li et al. in a recent study [7] also found a high rate of false-negative RT-PCR results, which were tested on specimens collected from 610 hospitalised patients from Wuhan, China. Thus, using RT-PCR as a reference standard would constitute using an imperfect gold standard. This would never let the other test show a better diagnostic performance. The additional patients identified would be regarded as false positives. With the use of chest computed tomography (CT) scans as a diagnostic method, the same mistake has also been made [8,9].In such a scenario, instead of calculating the sensitivity, specificity, and predictive values of the test (which would require one of the tests to be the gold standard), the best strategy would be to measure the degree of agreement (using the Kappa coefficient) between the two tests [10], i.e., neither of the two tests is considered to be the reference, and therefore, any discrepancies could be linked to either of the tests. Studies determining agreement between these tests are lacking in the literature. This study aims to estimate the agreement between RT-PCR and RAT for detecting severe acute respiratory syndrome coronavirus 2 (SARS-CoV-2), i.e., COVID-19 infection.

## Materials and methods

Study design, population, and setting

We conducted a retrospective record-based study between July 2021 and August 2021 after getting clearance from the Institutional Ethics Committee, Government Bundelkhand Medical College, Sagar, Madhya Pradesh, India (IECBMC/2021/24). Data was collected from the virology laboratory of the Government Bundelkhand Medical College from August 2020 to December 2020. It is prudent to mention here that vaccinations against COVID-19 in India started in January 2021. Hence, subjects in this study were unvaccinated for COVID-19. This tertiary care centre caters to a population from both urban and rural areas, and being a referral centre for COVID-19, it has facilities for both RT-PCR and rapid antigen tests. Patients, during their course of management, undergo both tests as per protocol. The study population consisted of people suspected of being infected by COVID-19 (who had symptoms) or contacts of confirmed cases (who could have been asymptomatic) identified through contact tracing.

Inclusion and exclusion criteria

We included only those subjects who had undergone both RT-PCR and RAT tests. The maximum acceptable duration between both tests was set to three days. We believe that three days is short enough to not have affected the severity of the condition and hence the diagnosis. The subjects and investigators were blinded to the result of the first test in cases where the second test was RAT. In those instances where RAT was conducted initially, it was not possible to blind the participant. Those subjects with data showing faulty sample collection (from sites other than the prescribed site) were excluded. Standard procedures were followed for conducting RT-PCR and RAT as recommended by the ICMR.

Kits used for RT-PCR and RAT

The kit used for RAT was the STANDARD Q COVID-19 Ag detection kit (SD Biosensor Inc., South Korea), and for RT-PCR, VIRALDTECT-II (Genes2Me Pvt. Ltd., India). All RT-PCR samples were oropharyngeal and all RAT samples were nasopharyngeal, as recommended by the manufacturer.

Sample size and sampling

The minimum required sample size was calculated using the formula for determining Cohen’s kappa, where we took the proportion of positive rating by rater one (i.e., RT-PCR) as 68% (sensitivity) [4], the proportion of positive rating by rater two (i.e., RAT) as 50% (sensitivity) [3], and assumed κ=85% at 95% CI with 10% precision. It was calculated to be 93. We took 100 subjects. Universal sampling was done, and subjects were included or excluded as per the criteria set above.

Study variables and data analysis

Data were abstracted in a data abstraction sheet designed with the help of Epi Info software (version 7.2.4.0, CDC, Atlanta) for Windows. We abstracted data on results of RT-PCR and RAT (positive and negative), age, sex, the test kit used, the time duration between samples taken for RT-PCR and RAT, and the presence of symptoms (cough, fever, sore throat, and generalised body ache) from the records of subjects for further analysis. Participants were classified as "symptomatic" if any of the symptoms were present. We used IBM Statistical Package for Social Sciences (SPSS) software (IBM Corp., released 2019. IBM SPSS Statistics for Windows, Version 26.0. Armonk, NY: IBM Corp.) for data analysis. Qualitative variables were summarised using percentages, and quantitative variables were summarised using mean (SD) and median (IQR). Keeping in mind the binary outcome of RT-PCR and RAT, Cohen’s Kappa Coefficient (κ) and agreement percentage (accuracy) were calculated. Kappa was classified as <0.00: poor, 0.00-0.20: slight agreement, 0.21-0.40: fair agreement, 0.41-0.60: moderate agreement, 0.61-0.80: substantial agreement, and 0.81-1.00: almost perfect agreement [11]. P-values <0.05 were considered significant throughout. All digital records were password protected, and only the chief investigator had access to them. The reporting was done as per the guidelines for reporting reliability and agreement studies (GRRAS) checklist [12].

## Results

We shortlisted 124 subjects, out of which 24 were excluded owing to incomplete information or other lacunae. Data on 100 subjects were finalised for analysis, which met all the inclusion and exclusion criteria. Fifty-eight (58%) were males and 42 (42%) were females, with ages ranging from two to 88 years. The mean age was 39.8 (SD ±19.0) years; the median age was 39 years (IQR 25.5-55 years).

Out of 100 subjects, 13 tested positive and 87 tested negative on both RT-PCR and RAT, with eight discordant pairs. Cohen’s kappa showed substantial agreement, κ=0.646, (95% CI 0.420 to 0.871), p<0.001 (Table 1).

**Table 1:** Performance of RT-PCR and RAT (n=100)

RAT result	RT-PCR		Total	Kappa (95% CI)	P value
	Positive	Negative			
RAT Positive	9	4	13	0.646 (0.420 - 0.871)	<0.001
RAT Negative	4	83	87		
Total	13	87	100		

**Figure 1:**
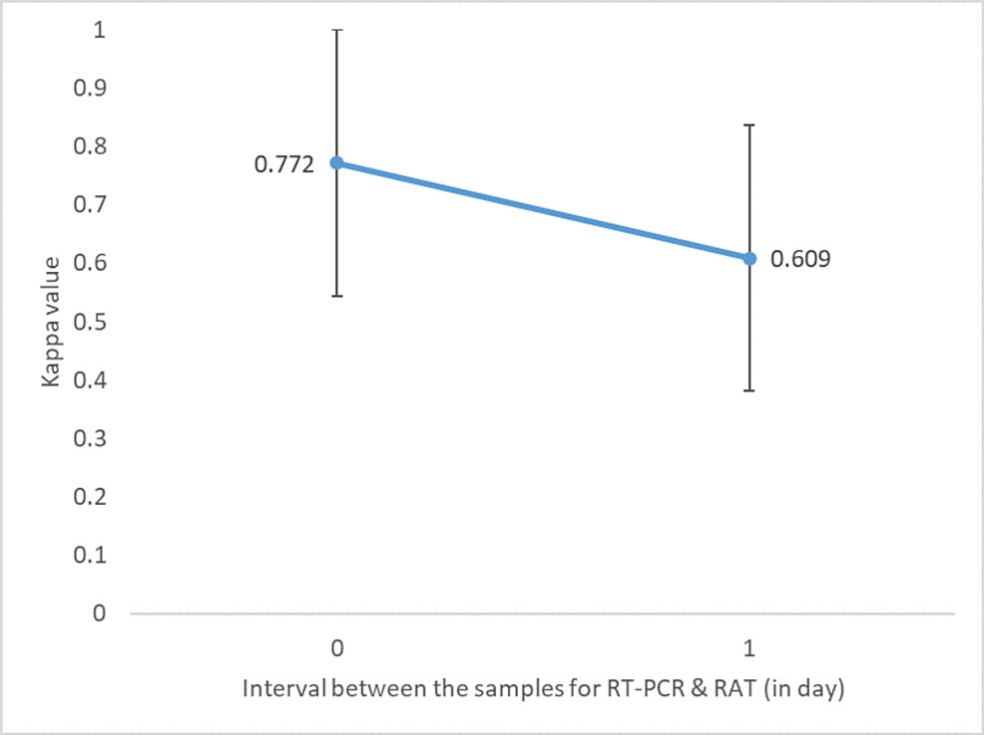
Variation in agreement vis-à-vis the time difference between samples of RT-PCR and RAT For a zero-day interval: N=61 and for a one-day interval: N=27

The Kappa varied with the intervals between taking samples for tests. Figure 1 shows this variation when the sample was collected on the same day and with a one-day difference, with accuracies of 95.1% and 88.9%, respectively. Both were statistically significant, with p-values <0.000 and <0.001 respectively.

When stratified based on the presence or absence of symptoms, 31 participants were symptomatic, having at least one of the symptoms, and 69 were asymptomatic. The corresponding kappa values were κ=0.59 (95% CI 0.28 to 0.91), p<0.001 and κ=0.64 (95% CI 0.27 to 1.0), p<0.001, showing moderate and substantial agreement among symptomatic and asymptomatic participants, respectively.

On RT-PCR, out of 13 participants who tested positive and 87 who tested negative, respectively, nine (69.2%) and 22 (25.3%) were symptomatic. Likewise, on the RAT, out of 13 participants who tested positive and 87 who tested negative, the number of symptomatic participants was eight (61.5%) and 23 (26.4%), respectively.

## Discussion

This study investigated the agreement between these two tests for SARS-CoV-2 detection with the help of Cohen’s Kappa [11]. Since the commencement of RAT testing in India, few reliable kits have been available. At that time, an independent two-site study of the sole available or stand-alone antigen detection assay in India, STANDARD Q COVID-19 Ag detection kit, was performed to assess its sensitivity, specificity, and practicality of application as a point-of-care test for early detection of SARS-CoV-2. However, the agreement between the two tests was not tested or mentioned. There are very few studies that have evaluated the agreement between these two tests. The majority of the papers available in the literature have evaluated diagnostic accuracy mainly using sensitivity and specificity [13-16]. With a Cohen’s kappa, κ=0.646, (95% CI 0.420 to 0.871), p<0.001, our study showed a substantial agreement between the two tests.

Jääskeläinen et al. [17] evaluated the performance of three rapid diagnostic tests (RDTs), viz., Sofia, STANDARD Q and Panbio™ and found overall agreements of 84.57%, 84.85%, and 86.32%, respectively, with a kappa value of 0.636, 0.633, and 0.660, respectively. This is similar to the results of our study on the STANDARD Q COVID-19 Ag detection kit. The study by Peña M et al. [18] on 854 asymptomatic individuals from the Chilean region calculated an accuracy of 97.04% with a kappa value of 0.78 (95% CI 0.70-0.86), which showed better performance than that of our study. Felipe Pérez‐García et al. [19] did a retrospective comparative evaluation between two antigen rapid diagnostic tests (Ag-RDTs), viz., Panbio and SD-Biosensor, for detection of SARS-CoV-2 and found agreement in accuracy and κ values of 80.9% and 0.596 and 82.6% and 0.646, respectively. The results were similar to our study. They also concluded that RDTs were excellent for the diagnosis of high viral load samples and those early in the disease.

Ristić M et al. [20] evaluated the clinical performance of the STANDARD Q COVID-19 Ag Test (SD Biosensor, Gyeonggi-do, South Korea) by analysing prospectively collected data on 120 symptomatic patients and concluded a strong agreement between the STANDARD Q COVID-19 Ag Test and RT-PCR with a kappa of 0.852 and a pooled accuracy of 92%, whereas our study reported a similar agreement percentage and a κ=0.59 (95% CI 0.28 to 0.91), p<0.001 among symptomatic patients. This difference could be explained by the fact that they included suspects who were in their first five days of illness, whereas our sample included a mix of suspects who had samples taken on various days after symptoms began.

Among the studies done in India, the strength of agreement between these two tests ranged from moderate to almost perfect. A prospective study [21] done among 756 patients from a district hospital in North India shows a substantial agreement like ours with a κ value of 0.6482 (95% CI: 0.5801 to 0.7163). However, Pandey et al. found moderate agreement between these two tests [22]. The reported κ value in their retrospective study was 0.57. Whereas, a κ value of 0.86 was reported by Gupta et al. in their study, which showed almost perfect agreement between RAT and RT-PCR [23].

In contrast, findings by Amer RM et al. in their cross-sectional study [24] on 83 COVID suspects showed much less agreement with a kappa of 0.3 (95% CI 0.16-0.59) and an accuracy of 75.9%, recommending against using RAT alone for COVID-19 diagnosis. With a smaller sample size in the Amer RM study, this sharp contrast between results needs to be interpreted carefully.

Limitations

Owing to the pandemic, logistical hurdles, and the retrospective nature of the study, we did not have control over the quality of samples for both tests. However, we considered only those participants for whom the sample was taken from the recommended site as mentioned by the kit manufacturer. We also could not consider the order in which the tests were conducted. However, we did take into account the time difference (in days) between collecting samples for the respective tests. The time interval between symptom onset and sample collection could not be recorded, even though the number of subjects included in the analysis is more than the minimum required sample size calculated with the given precision. However, evidence from a larger sample study would have been more conclusive.

## Conclusions

Evaluating the ease of conducting tests, the quick results, the lack of requiring a laboratory setup, the affordability, and the substantial agreement (κ=0.646, 95% CI 0.420 to 0.871, 92% agreement accuracy) with RT-PCR, we conclude that RAT may be a better choice for detecting SARS-CoV-2 and hence COVID-19 disease on a large scale. However, larger prospective studies would be needed to substantiate our findings.
